# Knowledge, attitude, and practice about rabies prevention and control: A community survey in Nepal

**DOI:** 10.14202/vetworld.2021.933-942

**Published:** 2021-04-17

**Authors:** Pushkar Pal, Adisorn Yawongsa, Tej Narayan Bhusal, Rajendra Bashyal, Theera Rukkwamsuk

**Affiliations:** 1 Department of Large Animal and Wildlife Clinical Sciences, Faculty of Veterinary Medicine, Kasetsart University, Nakhon Pathom 73140, Thailand; 2 Department of Veterinary Pathology and Clinics, Agriculture and Forestry University, Nepal; 3 Department of Plant Breeding and Genetics, Agriculture and Forestry University, Nepal; 4 Department of Anatomy, Physiology and Biochemistry, Agriculture and Forestry University, Nepal

**Keywords:** communities, developmental zones, knowledge, attitude and practice survey, Nepal, rabies

## Abstract

**Background and Aim:**

Rabies is a fatal zoonosis caused by RNA virus belonging to genus Lyssavirus. Nepal is one of the endemic countries in South Asia for rabies. This study was conducted to better understand the knowledge, attitude, and practice (KAP) of Nepalese community toward rabies across five developmental zones of the country.

**Materials and Methods:**

The cross-sectional study was carried out by face-to-face interview using structured questionnaires among 5000 respondents of five cities representing each of the five developmental regions of Nepal by adopting random cluster sampling procedure. The respondents were classified into four categories, including gender, age, education, and social status. The responses for KAP variables were analyzed using descriptive and Chi-square test.

**Results:**

The male and younger age respondents with higher education and social status were found more knowledgeable than their counterparts in terms of knowledge variables, including cause of rabies, mode of transmission, clinical signs, treatment, and preventive measures of this fatal disease. Similar findings were observed for attitude and practice variables such as vaccination practice, dog sterilization, health-seeking behavior, first aid practice, and use of first aid materials after dog bites. Some respondents in elderly age group still preferred to use traditional and local methods, which were application of turmeric powder and shrubs to cure dog bites rather than seeking medical facilities.

**Conclusion:**

There is a strong need for rabies awareness programs in the community targeting females, school, and college-level students, older age groups, and economically marginalized communities. The awareness materials need to focus on particular topics such as the risk of rabies, modes of transmission, the importance of first aid, health-seeking behavior following dog bite injuries, and practice preventive measures for their pets and community dogs.

## Introduction

Rabies, caused by a negative-stranded RNA virus within the Lyssavirus genus of the Rhabdoviridae family, is one of the oldest diseases accounting for significant public health issues [1]. The disease can be lethal to humans and animals once clinical symptoms have developed [2]. Although the disease is preventable, approximately 59000 people died of rabies every year, with 95% of these cases occurring in the Asian and African regions [3]. Despite considerable efforts, including extended control plans and general awareness programs to contain rabies infection, over 35000 cases still occur in Asia [1]. The primary source of animal and human rabies in the Asian continent is the bite of rabies-infected dogs, causing up to 95% of cases [4].

**Figure-1:**
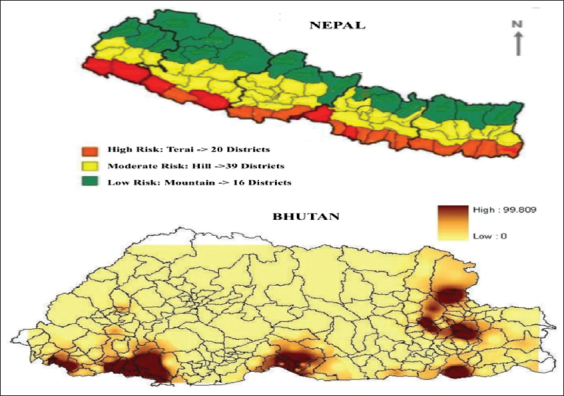
Rabies cross-border issue [Source: Annual Bulletin of Dogs Trust-2014].

Nepal is a small Himalayan country in South Asia. The Nepal’s southern plain areas, connected to the Indian border, grant restriction-free movement of animals and human beings, which causes rabies to be endemic in both countries [5]. A report described the open border facilitates in infection transmission, including rabies [6]. A similar report from Bhutan described that the higher number of rabies cases were recorded in the areas bordered with India [7]. A study carried out by Dog’s Trust, UK, in 2014, reported a higher number of rabies cases in Indo-Bhutan and Indo-Nepal borders than in the other parts of both countries (Figure-1). Rabies has rapidly become a highly relevant subject area for research; due to frequent outbreaks typically affecting domestic animals and humans. A study conducted in 2011 revealed that one hundred and fifty human deaths and a similar number of livestock deaths occurred every year in Nepal [8]. According to that study, the situation of rabies in the South Asian Association of Regional Cooperation is summarized in Table-1. In Nepal, the deaths resulting from rabies are likely to be underreported due to its topography, weak surveillance system, and inadequate knowledge about rabies [6]. There is a strong need for evidence-based research on epidemiology of rabies and community knowledge, attitude, and practice (KAP) toward rabies to plan the intervention programs of this disease [9].

**Table-1:** Situation of rabies in Nepal at South Asian Association of Regional Cooperation Region.

Countries	Estimated cases	Human cases, per 100000	% of dog bite
Afghanistan	2000-3000	5.7	N/A
Bangladesh	1500-2000	1.5	95
Bhutan	<10	0.28	99
India	18000-20000	3	>95
Nepal	150	0.21	98.5
Sri Lanka	<50	0.26	95
Pakistan	2000-5000	1.3	>90

Source: WHO, 2013

The KAP study has been extensively used in communities to understand their status toward infectious diseases across the world. The KAP study has also become helpful in comprehending the disease burden [10]. Moreover, the KAP study has become an integral part of the rabies control and elimination in both developed and developing worlds. In recent years, the Government of Nepal (GoN) has shown solidarity to eliminate rabies by 2030. However, there is no concrete policy and program based on the research. Although rabies is well known as an infectious disease and a burden in Nepal, it has not received significant attention for research. Furthermore, there is a lack of clarity on the available data due to limited studies [11].

Therefore, this study was designed to better understanding the community’s knowledge, attitude and practice about rabies in Nepal. These study findings would help concerned agencies, mainly the GoN, to effectively build up the rabies control plans.

## Materials and Methods

### Ethical approval

Ethical approval was not necessary for this study. However, individual consent was taken from the respondents who participated in the study.

### Study design, period and area

**Figure-2:**
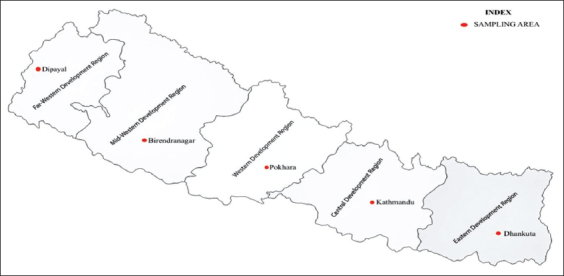
The sampling locations of knowledge-attitude-practice study.

A community-based cross-sectional survey was conducted from November 2018 to August 2019 in the five developmental zones of Nepal (recently changed to states), namely: Eastern, Central, West, Mid-west, and Far-west. The study was performed in the headquarter districts of respective developmental regions (Figure-2), inhabiting 602,733 people (2.46% of Nepalese population according to 2011 national census). These study areas have been assumed to be more advanced in terms of education, physical facilities, and human resource indices in their respective regions.

### Survey method

The study surveyed 5000 participants. One thousand participants (500 males and 500 females) were randomly selected from each of the developmental regions for the face-to-face interview. The random cluster sampling method was adopted for this purpose. The survey participants were categorized into gender, age, level of education, and social status. The study had a uniform sample population in the gender category and heterogeneous population in the remaining categories. Table-2 presents the demographic characteristics of our study population.

**Table-2:** Demographic characteristic of the survey respondents.

Variables	Characteristics	n (%)
Gender	Male	2500 (50.0)
	Female	2500 (50.0)
Age group	≤35	3130 (62.6)
	≥36	1870 (37.4)
Education level	School/college	4097 (81.9)
	University	903 (18.1)
Socio-economic status	Upper/middle	2265 (45.3)
	Lower	2735 (54.7)

### Sampling and interview procedure of households

To assess the KAP, the selected locations were further divided into sub-areas with the help of local village leaders and residents. The residential areas were divided into neighborhoods based on the household’s median income and education level. The KAP study was then conducted in each neighborhood. A door-to-door survey was conducted using a rolling sample method [12]. According to this method, the first selected household provides information about the next available house in the area or within the neighborhood, until the target number of household respondents was interviewed in the study area. However, it was guaranteed that representative samples were selected from all the selected locations. One adult person (>18 years of age) from each chosen household/family was interviewed face-to-face either in the morning or evening. The selected person was informed about the purpose of the study and that participation was voluntary. Data received from the respondents would be kept confidential. The interview was administered in the national language, and later the answers were translated into English.

### Questionnaire design

A well-structured questionnaire was designed to assess the KAP about rabies. The questionnaire was partly adapted from similar studies conducted previously [12,13]. Tables-3 and 4 demonstrate the determinants of KAP.

**Table-3:** Assessment of rabies knowledge.

Variables	Response
Cause of rabies	Microorganism
	Non microorganisms
Ways of transmission	Cat
	Dog
	Wild animals
	Rodents
	No answer
What are the symptoms of rabies	Salivation
	Altered personality
	Hydrophobia
	Aerophobia
	Don’t know
Is the disease treatable	Yes
	No
Is the disease fatal	Yes
	No
Is the disease preventable	Yes
	No

**Table-4:** Assessment of attitude and practice toward rabies.

Variables	Response
Have you heard about rabies?	Yes
	No
Do you vaccinate your pets/community dogs against rabies?	Yes
	No
Are you familiar with the vaccination/ABC^a^ campaigns organized by government?/Non-profits for Free-roaming dogs (FRD^b^)?	Yes
	No
What would you do when you get bitten by rabid animals/wild lives/pets	Seek medical care
	Traditional healer
	Do nothing
Do you practice first aid after getting bitten by rabid animals?	Yes
	No
Which first aid materials do you practice?	Soap water
	Herbal products
	Traditional methods

a Animal birth control,

b Free-roaming dog

### Data management and analysis

The questionnaire responses were transferred to Excel (Microsoft Excel, 2010, Microsoft Corp., Redmond, WA, USA) and made compatible with subsequent analysis using R studio. A Chi-square test was performed to test the association of the studied variables related to KAP. Odds ratios were calculated to describe the association of each variable (gender, age group, education level, and socio-economic status) and the questionnaire outcomes for KAP. Similarly, descriptive analysis was also performed to analyze the multiple responses.

## Results

### Knowledge about rabies transmission

The questionnaire had multiple choice answers to assess the knowledge on mode of transmission (Table-5). The descriptive analysis discovered that 55-70% of the respondents had a knowledge about the possible transmission route of rabies by the dog. However, all respondents had fairly less knowledge on other ways of rabies transmission. This finding would suggest that respondents had low level of knowledge about the ways of rabies transmission other than dogs. This knowledge gap in communities could be a risk factor for disease control programs.

**Table-5:** Multiple choice responses (%) about rabies transmission.

Transmission	Gender		Age		Education		Socio-economic status	
			
	Male	Female	≤35	≥36	School/college	University	Lower	Upper/Middle
Dog	65.4	58.88	58.68	68.05	62.52	60.03	57.91	68.35
Cat	15.04	17.4	17.49	10.57	14.10	20.72	16.67	16.07
Wild animals	6.52	7.44	8.18	5.51	5.49	12.30	8.48	4.76
Rodents	6.24	5.72	4.33	9.71	6.12	6.95	6.21	6.18
Don’t know	6.80	10.56	11.23	6.18	11.67	-	11.80	4.61

### Knowledge about rabies symptoms

The study discovered that the knowledge on clinical signs were meager in all the studied respondents (Table-6). The responses received for the altered personality, salivation, and hydrophobia, do not know and aerophobia were 36-48%, 29-37%, 6-19%, 5-16%, and 0.8-7%, respectively. The study confirmed that the community had very less knowledge concerning the clinical manifestation of rabies. Our findings hinted that the gap in the knowledge about clinical signs could be considered as a potential risk factor for the rabies elimination programs.

**Table-6:** Multiple choice responses (%) about rabies symptoms.

Transmission	Gender		Age		Education		Socio-economic status	
			
	Male	Female	≤35	≥36	School/college	University	Lower	Upper/Middle
Salivation	33.28	32.84	33.84	29.15	31.84	37.18	37.09	31.94
Hydrophobia	11.08	5.80	6.64	6.76	5.85	17.76	6.14	19.23
Aerophobia	2.28	0.84	1.65	1.71	0.75	4.44	0.58	6.98
Altered personality	45.00	44.12	45.47	43.53	48.25	30.80	44.20	36.38
Don’t know	16.40	16.40	12.37	14.28	13.27	9.80	18.46	5.43

### Analysis on knowledge about rabies according to gender and age

Analysis of responses relating to rabies knowledge among various determinant variables is presented in Table-7. Female populations were less likely to know about the determinants of rabies knowledge than their counterparts. Similarly, older (≥36 years) respondents were less knowledgeable than the younger group.

**Table-7:** Association of gender or age and the responses toward rabies knowledge.

Variables	Number	Gender		χ^2^-value	p-value	Age		χ^2^-value	p-value
	
		Male	Female			≤35	≥36		
Availability of treatment				36.35	<0.001			745.06	<0.001
Yes	3561	1877	1684			2652	909		
No	1439	623	816			478	961		
OR (95% CI)		1.0	0.68 (0.61-0.77)			1.0	0.17 (0.15-0.19)		
Rabies is fatal				13.59	0.0002			924.17	<0.001
Yes	3778	1945	1833			2812	966		
No/don’t know	1222	555	667			318	904		
OR (95% CI)		1.0	0.78 (0.69-0.89)			1.0	0.12 (0.10-0.14)		
Preventive measures				8.64	0.003			764.59	<0.001
Yes	4279	2176	2103			3011	1268		
No	721	324	397			119	602		
OR (95% CI)		1.0	0.79 (0.67-0.92)			1.0	0.08 (0.07-0.10)		

CI=Confidence interval, OR=Odds ratio

### Analysis on knowledge about rabies according to education and socio-economic status

In this category, respondents with university-level education were more knowledgeable than the respondents with school/college level education (Table-8). Similarly, respondents from upper/middle class were more familiar about the rabies knowledge than those from the lower-class participants. The study confirmed that rabies knowledge is influenced by the level of education and social class.

**Table-8:** Association of education or socio-economic status and the responses on rabies knowledge.

Variables	Number	Education		χ^2^-value	p-value	Socio-economic status		χ^2^-value	p-value
	
		School/college	University			Lower	Upper/middle		
Availability of treatment				219.45	<0.001			345.85	<0.001
Yes	3561	2674	887			1645	1916		
No	1439	1345	94			1081	358		
OR (95% CI)		1.0	4.73 (3.81-5.95)			1.0	3.52 (3.07-4.03)		
Rabies is fatal				2.03	0.15			242.54	<0.001
Yes	3778	2979	799			1831	1947		
No/don’t know	1222	940	282			904	318		
OR (95% CI)		1.0	0.89 (0.77-1.04)			1.0	3.02 (2.62-3.49)		
Preventive measures				3.78	0.05			63.60	<0.001
Yes	4279	3334	945			2242	2037		
No	721	585	136			493	228		
OR (95% CI)		1.0	1.22 (1.00-1.49)			1.0	1.96 (1.66-2.33)		

CI=Confidence interval, OR=Odds ratio

### Analysis of attitude and practice according to gender and age

The present study confirmed the findings of the attitude and practices of respondents (Table-9). Both male and female respondents almost equally vaccinated their pets/community dogs against rabies, sought medical care when bitten by suspected rabid animals, and used soap water to wash bite-wound. In the age category, older respondents were less likely to have a positive attitude and good practices including vaccinated their pets/community dogs against rabies, sought medical care when bitten by rabid animals, did first aid practice, and used soap water to wash bite-wound. In contrast, they were more likely to familiar with the vaccination/Animal birth control (ABC) campaign for free-roaming dogs (FRD) organized by the government or non-profits organizations. From these results, it was clear that the older age group seemed to have poor practice and attitude toward rabies compared to the lower age group. For successful elimination and control programs, all the community segments should adopt an appropriate attitude and practice toward the recommended measures.

**Table-9:** Association of gender and age and the responses relating to attitude and practices about rabies.

Variables	Number	Gender		χ^2^value	p-value	Age		χ^2^value	p-value
	
		Male	Female			≤35	≥36		
Hear of rabies				19.85	<0.001			36.72	<0.001
Yes	4672	2375	2297			2976	1696		
No	328	125	203			154	174		
OR (95% CI)		1.0	0.60 (0.47-0.75)			1.0	0.50 (0.40-0.63)		
Vaccination of own pets/community dogs against rabies				2.54	0.11			147.08	<0.001
Yes	3353	1703	1650			2294	1059		
No	1647	797	850			836	811		
OR (95% CI)		1.0	0.91 (0.81-1.02)			1.0	0.48 (0.42-0.54)		
Familiarity with vaccination/ABC^a^ campaigns for FRD^b^				6.67	0.01			36.11	<0.001
Yes	1294	687	607			720	574		
No	3706	1813	1893			2410	1296		
OR (95% CI)		1.0	0.85 (0.75-0.96)			1.0	1.48 (1.30-1.69)		
Medication practice when bite by rabid animals/wild lives				1.49	0.22			164.71	<0.001
Seek local healer	1427	694	733			695	732		
Seek medical care	3573	1806	1767			2435	1138		
OR (95% CI)		1.0	0.93 (0.82-1.05)			1.0	0.44 (0.39-0.50)		
First aid practice if bitten by rabid animals				0.52	0.47			328.01	<0.001
Yes	3595	1809	1786			2529	1066		
No	1405	691	714			601	804		
OR (95% CI)		1.0	0.96 (0.84-1.08)			1.0	0.32 (0.28-0.36)		
First aid materials in practice				0.04	0.84			138.07	<0.001
Soap water	3079	1543	1536			2123	956		
Others*	1921	957	964			1007	914		
OR (95% CI)		1.0	0.99 (0.88-1.11)			1.0	0.50 (0.44-0.56)		

* Herbal products or traditional methods,

a Anti-birth control,

b Free-roaming dog. CI=Confidence interval, OR=Odds ratio

### Analysis of attitude and practice according to ­education and status

Respondents with university-level education were more likely to be familiar with the determinant variables of attitude and practice than school and college respondents (Table-10). They heard of rabies, vaccinated their pets/community dogs against rabies, were familiar with the vaccination/ABC campaign for FRD, sought medical care when bitten by rabid animals, practiced first aid, and also equally used soap water to wash bite-wound by either of the education levels. Besides, respondents from the upper/middle class were more likely to be familiar with the attitudes and practices of rabies than their counterparts. They heard of rabies, vaccinated their pets/community dogs against rabies, were familiar with the vaccination/ABC campaign for FRD, sought medical care when bitten by rabid animals, did first aid practice, and used soap water to wash bite-wound. This result highlights that respondents with higher education and higher social status followed the standard practices and had better attitudes toward rabies than their counterparts.

**Table-10:** Association of education and socio-economic status and the responses relating to attitude and practices about rabies.

Variables	Number	Education		χ^2^-value	p-value	Socio-economic status		χ^2^-value	p-value
	
		School/College	University			Lower	Upper/Middle		
Hear of rabies				35.46	<0.001			172.8	<0.001
Yes	4672	3619	1053			2441	2231		
No	328	300	28			294	34		
OR (95% CI)		1.0	3.10 (2.13-4.70)			1.0	7.87 (5.57-11.48)		
Vaccination of own pets/community dogs against rabies				41.45	<0.001			38.08	<0.001
Yes	3353	2540	813			1732	1621		
No	1647	1379	268			1003	644		
OR (95% CI)		1.0	1.64 (1.41-1.92)			1.0	1.46 (1.29-1.64)		
Familiarity with vaccination/ABC^a^ campaigns for FRD^b^				165.96	<0.001			148.43	<0.001
Yes	1294	850	444			520	774		
No	3706	3069	637			2215	1491		
OR (95% CI)		1.0	2.52 (2.18-2.90)			1.0	2.21 (1.94-2.52)		
Medication practice when bite by rabid animals/wild lives				14.77	<0.001			47.39	<0.001
Seek local healer	1427	1169	258			890	537		
Seek medical care	3573	2750	823			1845	1728		
OR (95% CI)		1.0	1.36 (1.16-1.59)			1.0	1.55 (1.37-1.76)		
First aid practice if bitten by rabid animals				176.38	<0.001			292.23	<0.001
Yes	3595	2644	951			1696	1899		
No	1405	1275	130			1039	366		
OR (95% CI)		1.0	3.52 (2.91-4.30)			1.0	3.18 (2.78-3.64)		
First aid materials in practice				0.002	0.96			138.12	<0.001
Soap water	3079	2414	665			1483	1596		
Others*	1921	1505	416			1252	669		
OR (95% CI)		1.0	1.00 (0.87-1.14)			1.0	2.01 (1.79-2.27)		

* Herbal products or traditional methods,

a Anti-birth control,

b Free-roaming dog. CI=Confidence interval, OR=Odds ratio

## Discussion

This study aimed to comprehend the knowledge, attitudes, and practices of rabies among the communities of five developmental regions in Nepal. To the best of our knowledge and understanding, this was the first large-scale KAP study on rabies conducted in Nepal. This study has few limitations, such as non-random selection of respondents due to topography, the distribution of houses, and the unwillingness of residents to participate. However, the sample population had similar demographic characteristics similar to that of the general Nepalese population. The current study has demonstrated statistically significant differences in knowledge, attitude, and practice of respondents and has identified several knowledge gaps.

### Knowledge

This was surprising given the fact that all categories of respondents had very less knowledge about the cause of rabies. About 7% of the 275 participants answered correctly about the cause of rabies in India. This might be due to a higher level of education and awareness level of Indian community toward rabies [14]. However, this study was inconsistent with Sri Lanka’s study [13]. They reported that 86.2% of the respondents knew that rabies was caused by microorganisms and spread by dogs, which was found similar to an earlier study done in Gujarat [15]. Their findings indicated higher level of awareness and knowledge in the respective communities.

About understanding of ways of disease transmission, the response (ranged from 58.68 to 68.35%) had identified the dog as a possible vector for rabies transmission. However, the rest of population responded in a mixed, but minimal way such as wildlife, rodents, and cats, were identified as vehicles for rabies transmission. These findings were similar to that of a study done in Ethiopia [16]. According to their study, high school respondents (58%) and higher education respondents (73%) positively responded toward ways of transmission and clinical symptoms. Conversely, this finding is lower than the studies conducted elsewhere in Tanzania [17] and in Namibia [18]. Our study obtained that a large segment of the population was completely unaware of the mode of transmission, which could be considered a significant knowledge gap. Such gaps in the knowledge series reflect the inconsistent awareness in all the age groups and genders [19]. The similar nature of the knowledge gap about the cause, ways of transmission, and clinical manifestation were also reported in Bhutan [20]. The parallel results between these two countries might be due to similar cultural beliefs toward the animals and similar education levels of the communities in both countries.

The response received from all groups for the prescribed clinical signs (altered personality, salivation, hydrophobia, and aerophobia) was lower than 50%. The results highlighted that the level of knowledge on clinical symptoms was deficient compared to other studies performed elsewhere in the world. One study in Ethiopia described that over 73% of participants were familiar about clinical symptoms of rabies [21]. These vast differences might be due to the intervention program administered by national and international health agencies in Ethiopia. In recent times, international agencies for rabies control programs are more focused in African regions than the Asian countries. The results suggest that there is a strong need of community awareness about rabies clinical signs.

Regarding the treatment of rabies, both males and females were less knowledgeable about the rabies treatment and its availability. The Sri Lankan study reported that above 93% of the community, mainly female participants, were well informed about the rabies treatment and prevention procedures [22]. This might be due to multiple interventions in rabies control programs in Sri Lanka, and such activities were lacking in Nepal. The respondents with university-level education were more likely to know the rabies treatment and its availability of treatment of rabies than their counterparts. Similarly, we observed that the upper/middle class was more knowledgeable about the treatment of rabies than the lower social class. Closer examination of the results revealed that the participants with a better level of education and higher social status had better knowledge about rabies treatment and its availability. Thus, participants from lower social strata and lower education could be risk factors for disease control. This finding strongly agreed with the findings of Tiwari *et al*. [23]. The high level of knowledge about rabies treatment in higher education and upper-class populations might be due to their access to multiple information sources such as government campaigns, activities of non-profits organizations working toward ABC and rabies control, and mass media [22]. This also applies to the Nepalese context. Our study further tied well with the findings of Ntampaka *et al*. [24]. They also reported similar observations that the higher level of education might have helped participants to understand the treatment methods and their availability in their area.

For the fatality of rabies, another variable of the knowledge, the female population was less likely to know about the outcome of rabies compared to their counterparts. Similarly, the old age group was found to have less knowledge of rabies fatality. This finding was consistent with the reported from Sri Lanka[13]. However, this study’s results were in contrast with the previous study performed in a community of Tanzania [25]. Likewise, the upper/middle-class respondents were better informed about rabies’ fatality than the lower social status group. They indicated the post-exposure prophylaxis (PEP) and use of rabies immunoglobulin (RIG) in the case of rabies suspected animal bites.

Concerning knowledge on rabies preventive measures, the female population was found to have less knowledge than male participants. In the same way, the analysis revealed that the older age (≥36 years) was less likely to know preventive measures. This study agreed with previous studies conducted elsewhere [26]. On the contrary to our findings, the previous studies from India [27,28] reported that female and older age populations were more knowledgeable than their counterparts. The country level’s differences might be due to their awareness priorities in the community population.

Likewise, the participants with university degrees displayed the higher level of knowledge than college and high school level students. In the same way, upper-class respondents responded well about the preventive measures of rabies than the lower social status group. This finding was comparable with the findings of the studies conducted elsewhere in Sri Lanka and India [22,29]. Both studies reported a similar observation regarding knowledge and prevention. This might be attributable to the high awareness and educational level of participants. We also found good agreement when comparing our results to other studies [30]. This study explained that the educated population and people with a high standard of living were more aware of rabies and vaccinated their pets because of their excellent education and financial status.

### Attitude and practice

This section highlights the community responses about people’s attitudes and behavioral practices regarding rabies. The respondents were interviewed to assess if they have heard about rabies, vaccinated their pets/strays, healthcare beliefs in case of dog bites, first aid practices, and type of first aid materials they preferred to use.

The majority of both genders responded that they have heard about the disease. This finding corresponded with the previous studies in Bhutan [13,20]. Both male and female respondents were almost equally aware of the rabies vaccination practice. This response of the participants indicated that they had a moderate level of information about the vaccination practice that could protect animals or humans from rabies. This might be due to the recent efforts on rabies control initiated by non-profit organizations and volunteer organizations in Nepal. Similar conclusions were made by the previous studies conducted in Sri Lanka and India [13,29]. Concerning the age group respondents about attitude and practice towards rabies, the people above 36 years old were less likely to know about rabies. These findings were compatible with the findings of the previous studies performed elsewhere in Sri Lanka and Bhutan [20,22]. They mentioned that the younger generation was much smarter than the age group above 40 years old because of their education, social contacts, and access to media and technology. This study went beyond the previous study conducted in Tanzania [17]. The majority of the younger age group (96%) of the test population had heard about rabies.

The sample population in the category of higher education and higher level of social status corresponded positively with the attitude and practice variables. University-level education and high social status respondents have heard of rabies. These findings were similar to that of a study done in Ethiopia [16]. According to their study, high school respondents (58%), higher education respondents (83%), and upper/middle class of people in the society positively responded that they have heard about rabies. Conversely, this finding was lower than the studies conducted elsewhere in Tanzania [17] and in Ethiopia [31]. However, both studies agreed with the participants’ response that the attitude and practice toward rabies were higher with the advanced level of education and class of the society [32].

Regarding health-seeking behavior in dog bites, our study revealed that the male population is more likely to contact the medical facilities in case of dog bites. More recent studies demonstrated higher proportion (84%) of the male respondents in India [27], 97% of the males in Bali, Indonesia [33] and 94% in Philippines [30] used to go to the hospital in case if they got the dog bites. The medical-seeking behavior in neighboring countries seemed to be higher than Nepal. It might be due to the higher level of knowledge, education, and financial status of those countries. Moreover, in our study, it was quite a surprise to find that some respondents in both genders used to go to the local healers for a dog bite treatment instead of hospitals. Few of them preferred to use herbal products rather than consulting medics. Most of the time, these herbal products cause harmful effects though they contain a certain amount of medicinal value [34]. We suggested that the medical-seeking behavior in the female population was lower than the male respondents. The traditional practices to treat the dog bite were superstitious and needed to be corrected as soon as possible. This conservative belief could be a risk factor in controlling the disease in a community. Similarly, the older age group was less likely to be interested in health-seeking behavior for the dog bite. This finding was in close agreement with other studies done elsewhere in Tanzania [25] and India [35]. Tanzanian study revealed that only 5% of the sample population used to go to the hospital for the treatment, and only 20.4% of the respondents were aware of the proper consultation and medical care. In this study, a small number of the respondents preferred to take treatment services from the local healers or apply herbal products over the wound including use of salts, spices, turmeric powder, chili powder, and plant materials, that were not scientifically recommended. The use of such a practice was always life-threatening [1]. The use of soap water, PEP, and infiltration of RIG around the bite could prevent human rabies [1]. However, in this study, over 60 years (84.33%) sample population preferred to go to local healers to manage the dog bites. This unscientific practice among old age people could be due to their low level of education and awareness.

In response to first aid practice, both genders were likely to have equal know-how about the practices. They used soap water to clean the dog bite wound. However, a limited number of respondents preferred to consult the local healer and applied the herbal products and spices as a substitute for the first aid. Washing the dog bite wound with soap water has been strongly recommended [1] Nepalese participants’ response was relatively better than the previous reports found in Rwanda, Tanzania, and India, Rwanda (20.4%), Tanzania (5%), and India (43.07%) [24,25,35]. Therefore, the study suggested that awareness and educational programs were required in the communities to control rabies. Use of herbal plants and other ingredients was not recommended, and could not replace the standard first aids.

In the same way, the study demonstrated that the older respondents had low-level awareness on first aid and their use. Our findings were in contrast to the previous study from Gujarat [12]. They observed a significantly higher number of people in the older age group practicing the first aid with the recommended materials (soap water) or other detergents such as iodine constituents or according to their availability. A similar study in India [36] showed that a majority of participants, including senior citizens, would practice the recommended procedures (soap water) to clean the wound. This might be due to the differences in the community KAP level of both countries’ communities. Indian communities were more advanced in terms of education and financial status than Nepalese society, which could have led them to understand rabies control practices better. However, in our study, the older group was more familiar with the ABC program conducted by government or non-profit organizations in their area. The older group might have received this positive perception through government agencies since the government officials made contact with senior residents in the village to get their support to conduct the ABC and vaccination programs in the communities.

Similarly, respondents (upper social class) were found to practice cleaning the wound using soap water or detergents. Other studies recorded that the KAP with rabies is far better in urban residents than rural communities [24]. A better understanding of rabies knowledge in the upper-class people and higher education level may be due to their education, social contact, and self-awareness level [37]. Similar observations were also reported from Ethiopia [26].

Likewise, attitude and practice about the vaccination against rabies, the analysis revealed that the older participants were less likely to practice vaccination in animals (dogs). This finding was in line with the finding of a study reported in the Philippines that the younger group was more aware and practiced the vaccination program in the owned or stray population of dogs [30]. This might be due to the opportunity the younger age people receive, such as interacting with public hearings/meetings, reading newspapers, or accessing online information [13]. In a similar fashion, higher education and the higher socio-economic group were found to vaccinate their pets/community dogs against rabies more regularly than the lower level of education and social status group. This study has shown that the community KAP level is subtle in Nepal except in the respondents with higher education and social status. There is also a significant knowledge gap in risk of rabies, its cure, transmission ways, symptoms, and post-bite management issues.

## Conclusion

This analysis leads to some useful conclusions. The KAP level was slightly higher in men and people with higher education and socio-economic status than their counterparts. However, most of the participants were unaware of disease transmission methods, clinical symptoms, and causative factors. Similarly, a significant population also prefers to consult traditional healers and use local remedies over using medical facilities to treat dog bites injuries. Hence, the most relevant conclusions were that there was a need for social awareness programs targeting females, school and college-level students, older age groups, and economically marginalized communities to control rabies in communities. The awareness materials needed to focus on areas such as the risk of rabies, ways of transmission, the importance of first aid, health-seeking behavior following animal bites injuries and practice preventive measures for their pets and community dogs around them.

## Authors’ Contributions

PP and TR conceived the study plan and prepared the manuscript. AY and TNB analyzed the data. RB participated in data collection. All authors read and approved the final manuscript.
